# Decoding the lncRNA landscape in bovine mastitis: insights into immune regulation and gene networks underlying host response

**DOI:** 10.1186/s40104-026-01500-0

**Published:** 2026-10-01

**Authors:** V. Bisutti, D. Giannuzzi, A. Vanzin, G. Giannotta, A. Cecchinato, G. Polizel, V. Asselstine, Á. Canovas, S. Pegolo

**Affiliations:** 1 DAFNAE, University of Padova, Viale Dell’Università 16, Legnaro, PD 35020 Italy; 2 Centre for Genetic Improvement of Livestock, Department of Animal Biosciences, University of Guelph, Guelph, ON N1G 2W1 Canada

**Keywords:** Bovine mastitis, Immune response, Long non-coding RNA

## Abstract

**Background:**

Long non-coding RNAs (lncRNAs) are critical regulatory elements of gene expression and play a crucial role in immune regulation of disease, such as bovine mastitis. With this study we evaluated expression profile of lncRNAs in subclinical intramammary infection (sIMI) from two pathogens: *Streptococcus agalactiae* (*S. agalactiae*; Sa+) and *Prototheca* spp. (P+) and predicted their interaction with protein coding genes in 31 Holstein cows. Quantitative trait loci (QTL) annotation and enrichment analysis was carried out on the lncRNAs partner genes for a comprehensive overview of their potential association with phenotypic traits. Finally, Partial Correlation and Information Theory (PCIT) and Regulatory Impact Factor (RIF) algorithms were adopted to assess co-expression network of differentially expressed lncRNAs (DELs) and genes (DEGs).

**Results:**

A total of 211, 146, 353 and 184 DELs were identified when comparing Sa+ vs. Neg, P+ vs. Neg, Sa+ vs. P+, and uninfected vs. infected animals (Mast vs. Neg), respectively. Most of these DELs were mainly located within genic regions, and 78% had not been previously annotated in the bovine reference genome. Overall *S. agalactiae* infection modulated both immune and metabolic pathways (lipid metabolism, extracellular matrix synthesis), whereas *Prototheca* spp. infection activated immune signaling networks (B-cell and innate immunity). Unique lncRNAs induced by Sa+ were predicted to target genes controlling fatty acid synthesis and milk fat QTLs, while P+-induced lncRNAs were found associated mostly with immune genes and somatic cell count QTLs. The co-expression network also revealed shared lncRNAs between the two types of infection suggesting a core response: both infections converge on common hub genes (e.g., *EBF1*, *BTLA*), indicating potential conserved host-defense mechanisms.

**Conclusions:**

These results suggest that, despite distinct lncRNA expression patterns, the downstream regulatory pathways—particularly those linked to immune function—may converge toward a shared host response during subclinical intramammary infections. Together, these findings underscore the complexity of lncRNA-mediated regulation in the bovine mammary gland and highlight the need for functional studies to clarify the biological roles of these novel transcripts.

**Supplementary Information:**

The online version contains supplementary material available at https://doi.org/10.1186/s40104-026-01500-0.

## Background

Mastitis, particularly in its subclinical form, represents not only a major health challenge in dairy herds but also a significant economic burden. Subclinical mastitis often goes undetected due to the absence of overt clinical signs, yet it leads to persistently elevated somatic cell counts, reduced milk quality and yield, and increased culling rate [1]. The hidden nature of subclinical infections allows them to persist unnoticed within the herd, posing a risk of widespread intramammary infection [2]. Besides economic losses, subclinical mastitis affects animal welfare by causing chronic inflammation and discomfort, which can impair overall health and longevity [3]. In addition, the use of antimicrobials to control mastitis, especially in cases where causative agents are challenging and the condition becomes recurrent or chronic, has contributed to the development and increase of antimicrobial resistance. These multifaceted challenges highlight the need for deeper understanding of the molecular mechanisms and pathways underlying pathogenesis, ultimately informing more effective prevention and control strategies. In this context, the regulation of gene expression by non-coding RNAs (ncRNAs) plays a critical role in determining the susceptibility or resistance of dairy cows to mastitis. Advances in high-throughput sequencing technologies have revealed that a large proportion of the genome is transcribed into a heterogeneous population of ncRNAs [4]. Among these, long non-coding RNAs (lncRNAs) are a class of transcripts longer than 200 nucleotides that constitute a highly functionally diverse class of regulatory elements in gene expression [5, 6]. Once regarded as genomic “dark matter”, lncRNAs are now recognized as critical regulators of gene expression through both transcriptional and post-transcriptional mechanisms, including chromatin modification and alternative splicing [7, 8]. Notably, lncRNAs influence inflammation by modulating cell differentiation, cell cycle regulation, as well as key signalling pathways, including JAK-STAT, MAPK, and NF-κB [9]. Given their involvement in immune regulation and disease, lncRNAs could have a central role in the host's defense mechanisms of mastitis. Several in vitro studies have challenged mammary epithelial cells with common mastitis pathogens, such as *Staphylococcus aureus*, *Escherichia coli*, and lipopolysaccharide, demonstrating differential lncRNA expression in response to these stimuli [10, 11]. Particularly, Asselstine et al. [12] evaluated bovine somatic cells transcriptome for the detection of lncRNAs differentially expressed in healthy and mastitic cows. However, these approaches often lack precise pathogen-level resolution, limiting our understanding of the specific host responses involved.

To address this limitation, our study incorporates detailed bacteriological diagnosis to identify the causative agents of mastitis, including *Streptococcus agalactiae* and *Prototheca* spp., an emerging algal pathogen. Notably, *Prototheca* is resistant to conventional antibiotic treatments and it is often difficult to detect [13]. Given the emerging nature of *Prototheca* and the lack of effective treatment options, lncRNAs may offer new insights into host susceptibility or resistance mechanisms. We evaluated the expression profiles of lncRNAs using RNA-Sequencing (RNA-Seq) in the bovine mammary gland during naturally occurring subclinical intramammary infection (sIMI) and predicted their potential interactions with target protein-coding genes in Holstein cows. By combining integrative transcriptomic functional data with pathogen identification, our study seeks to uncover lncRNA-mediated regulatory networks that could contribute to the development of alternative diagnostic markers or genetic selection strategies to improve mastitis resilience in dairy cattle.

## Materials and methods

### Animal data

The study involved 31 Holstein cows from which 31 individual milk samples were collected manually and aseptically. These animals were selected from a commercial dairy herd of 450 lactating cows (Veneto region, Italy) regularly monitored by the Istituto Zooprofilattico Sperimentale delle Venezie (IZSVe) for the presence of *S. agalactiae* and *Prototheca* spp. For the study participation we required animals to fulfil the following criteria: i) absence of clinical signs of infection (i.e., only subclinical cases were included in this study) including no redness, swelling or palpable abnormalities of the udder, and no visible changes in milk appearance), ii) no antibiotic or anti-inflammatory treatments in the 3 months before the enrolment, iii) being multiparous, non-pregnant and at least 98 d in milk (DIM). These criteria were included to reduce to a minimum any potential sources of variation that could affect the outcome of the study. Moreover, animals assigned to the healthy group had no previous history of mastitis. Considering these criteria, a first bacteriological screening (time 0, T0) was conducted on a cohort of 188 animals to firstly identify healthy individuals and cows with sIMI from *S. agalactiae* (Sa+) and *Prototheca* spp. (P+). To confirm the infection status of the animals, a second bacteriological examination (T1) was conducted two weeks after T0. We then defined three experimental groups according to the outcome of the microbial analysis conducted at T0 and T1 as follows: i) healthy cows (*n* = 9) with negative bacteriological examination in all glands at T0 and T1; ii) animals naturally infected by *S. agalactiae* (*n* = 11), and iii) animals naturally infected by *Prototheca* spp. (*n* = 11). For the infected animals, the attribution of their health status was given if milk from at least one mammary gland quarter was positive for either *S. agalactiae* or *Prototheca* spp. Animals with co-infections were excluded from the trial.

All cows were managed under identical conditions and fed total mixed ration mainly based on corn silage, sorghum silage, and concentrates. The detailed description of the experimental plan, management of the animals and feeding conditions are reported by Pegolo et al. [14] and Bisutti et al. [15].

### Ethical statement

This research was approved by the Ethical Animal Care and Use Committee (OPBA - Organismo Preposto al Benessere degli Animali) of the Università Cattolica del Cuore and by the Italian Ministry of Health (protocol number 510/2019-PR of 19/07/2019).

### Collection of milk samples

Before the morning milking, milk samples were aseptically collected from all four mammary glands following the guidelines of the National Mastitis Council [16]. Briefly, teat ends were cleaned using a commercial pre-milking disinfectant, dried with individual towels, and subsequently disinfected with 70% alcohol. After discarding the initial streams of foremilk from each quarter, individual quarter-level milk samples were collected for microbiological analysis. Then, two additional 50 mL aliquots of composite milk, prepared by combining equal volumes of milk from all four mammary gland quarters, were collected for the determination of i) milk composition and somatic cell count (SCC) and ii) RNA extraction and transcriptomic profiling. Samples were stored at 4 °C upon arrival at the different laboratories.

### Microbiological analysis

Microbiological analysis was conducted by the IZSVe laboratory (Legnaro, PD, Italy). After collection, milk samples were transported to the laboratory, frozen at −20 °C and analysed within 3 d. Specifics of the adopted procedures and protocols are reported by Pegolo et al. [14]. Briefly, 10 μL of every sample were inoculated in the following selective media: (1) tallium kristalviolette tossin agar (IZSVe internal production) and (2) *Prototheca* isolation medium (PIM; IZSVe internal production). After 24 h of incubation, suspected colonies of *S. agalactiae* were confirmed using the Christie–Atkins–Munch-Peterson test [16]. After 24, 48, and 72 h, PIM plates were examined for the growth of *Prototheca*. Suspected colonies were confirmed by mass-spectrometry analysis [16]. Furthermore, a screening of the most common microorganisms responsible for mastitis, such as *S. aureus* and *S. uberis*, as well as some environmental ones, such as *Streptococcus* spp., *Staphylococcus* spp., *Klebsiella* spp., and *Enterococcus* spp., was conducted to avoid possible bias in the trial.

### Analysis of milk composition and udder health traits

Milk composition (protein, casein, lactose, fat, and urea content) were assessed on fresh samples using an FT6000 Milkoscan infrared analyzer (Foss A/S, Hillerød, Denmark), while SCC and DSCC were measured through the Fossomatic 7 DC analyzer (Foss A/S).

### RNA extraction and library preparation

RNA extraction from milk somatic cells and library preparation were performed following the workflow described in Bisutti et al. [15]. Briefly, the 31 RNA samples were sent to the Nuova Genetica Italiana (NGI, Como, Italy) facility for library preparation and sequencing. The RNA-Seq libraries were prepared from 500 ng of total RNA using the MGIEasy RNA Library Prep Set V3.1 (MGI Tech Co., Ltd., Shenzhen, China), according to the manufacturer’s protocol, after ribosomal rRNA removal using the MGIEasy rRNA Depletion kit V1.1 (MGI Tech Co., Ltd., Shenzhen, China). The RNA-seq analysis was performed on a DNBSEQ-G400 high throughput machine (MGI Tech Co., Ltd.) using a paired-end approach using the DNBSEQ-G400 sequencing kit (MGI Tech Co., Ltd., Shenzhen, China).

### Data mining

The quality of raw reads was assessed using FastQC (v0.12.1) [17], and adapter trimming as well as removal of low-quality reads was performed using Cutadapt (v5.0) [18]. Clean reads were then aligned to the *Bos taurus* ARS-UCD1.2 reference genome (Ensembl release) using HISAT2 (v2.2.1) [19]. Aligned reads were assembled into transcripts using Cufflinks, and sample-specific annotation files were generated. These individual annotation files were then merged using Cuffcompare to produce a single unified transcriptome annotation file (.gtf) representing all the assembled transcripts in the experiment [20].

The lncRNAs were identified using the FEELnc pipeline (https://github.com/tderrien/FEELnc) [21]. Three FEELnc modules were applied. First, FEELnc filter was used to remove transcripts annotated as protein-coding genes, pseudogenes, and miRNAs, and retain only transcripts ≥ 200 bp. Then, FEELnc codpot was employed to estimate the coding potential of each candidate transcript in the combined.gtf file. The coding potential score (CPS; range 0–1) was calculated based on k-mer frequencies (default: 1-, 2-, 3-, 6-, 9-, and 12-mers), ORF coverage, and mRNA size. FEELnc automatically determines an optimal classification threshold using the Two-Graph Receiver Operating Characteristic (TG-ROC) approach, which identifies the CPS corresponding to balanced sensitivity and specificity. In the present study, the optimal CPS was 0.573, at which sensitivity and specificity reached approximately 0.986. Transcripts with coding probabilities below this threshold were classified as non-coding, whereas transcripts with higher values were classified as coding. Finally, FEELnc classifier was used to identify the closest protein-coding gene associated with each lncRNA according to genomic context. Partner genes were defined based on *cis* positional relationships (overlapping, nested, containing, antisense, or other genic/intergenic configurations) between lncRNAs and neighboring protein-coding genes [12, 22, 23]. The lncRNAs were categorized as previously annotated when they had a specific Ensembl identifier. Expression quantification was performed using HTSeq-count (v2.0.9) [24] on two different matrices to obtain raw counts for each transcript and gene. Transcript-level quantification was used for lncRNAs to account for their isoform-specific expression patterns and potential functional diversity, providing higher biological resolution when investigating specific regulatory mechanisms. Normalization of the transcriptomic data with the sequencing depth was made for each sample by calculating counts per million (CPM). Then, transcripts with CPM < 0.5 and expressed in less than 10 samples were filtered out using the edgeR package, as also detailed in Bisutti et al. [15]. Differential expression analysis was then conducted separately on the gene and transcript matrices with a pairwise approach using DESeq2 R package (v1.49.0) [25] to identify both differentially expressed genes (DEGs) and transcripts (DETs). Specifically, the following comparisons were tested: i) *S. agalactiae* naturally infected animals vs. negative (Sa+ vs. Neg), ii) *Prototheca* spp. infected animals vs. healthy individuals (P+ vs. Neg), *S. agalactiae* vs. *Prototheca* spp. infected animals (Sa+ vs. P+), and iv) infected vs. uninfected animals (Mast vs. Neg), regardless of the pathogen. Lactation stages of the animals were included in the analysis as batch factor in the DESeq2 model. Parity was not included because exploratory analyses indicated a negligible effect of parity within the study population. DEGs and DETs were defined as those having a Benjamini and Hockberg corrected false discovery rate (FDR) < 0.01 and |log_2_ fold change| (|log_2_FC|) > 1. Finally, to identify differentially expressed lncRNAs (DELs) within each experimental group, the list of DETs obtained from the transcript-level DESeq2 analysis was intersected with the FEELnc output, which provided the annotated set of predicted lncRNAs.

### Quantitative trait loci (QTL) annotation and enrichment analysis

The QTL annotation and enrichment analyses were performed on all the partner genes of the identified DELs using the R package GALLO (Genomic Annotation in Livestock for Positional Candidate Loci, v. 1.5) [26], to obtain a comprehensive overview of their potential associations with phenotypic traits. Although not all DELs may exert regulatory functions, this exploratory approach allowed the identification of genomic regions potentially influenced by lncRNA activity. The analysis incorporated genomic coordinates of partner genes, along with the QTL.gff annotation file downloaded from the Cattle QTL Database (https://www.animalgenome.org/cgi-bin/QTLdb/index). For each lncRNA partner gene, QTL annotation considers a 1,000 bp interval upstream and downstream based on its genomic coordinates [27]. Chromosome-specific enrichment was evaluated using the qtl_enrich function to identify QTLs significantly overrepresented near the lncRNA partner genes. Multiple testing was controlled via FDR correction, with traits considered enriched at FDR < 0.05.

### Partial correlation and information theory and regulatory impact factor analyses

The list of DEGs and DELs obtained for each comparison were used to assess co-expression between lncRNAs and mRNAs. The construction of co-expression networks was carried out with the Partial Correlation and Information Theory (PCIT) algorithm within the CeTF v.1.16.0 R package [28]. This analysis incorporated all DELs and protein coding DEGs within each comparison (Sa+ vs. Neg, P+ vs. Neg*,* Sa+ vs. P+ *,* Mast vs. Neg cows) to ascertain the correlations among them, which were visualized in a network using the Cytoscape (v. 3.10.1) software. To facilitate the biological interpretation of the network and focus on high-confidence interactions, only associations with absolute correlation coefficients greater than 0.90 (*r* > |0.90|) were retained. Each experimental comparison generated two co-expression networks, for which their topological parameters and node centrality values were analyzed using the NetworkAnalyzer Cytoscape plugin to gain insights into the organization and structure of the complex networks formed by the interacting modules. The Molecular Complex Detection (MCODE) Cytoscape plug-in (v. 2.0.2) [29] was then adopted to identify the most densely connected modules within the interaction network. The cluster finding parameters included a degree cutoff of 5, a node score cutoff of 0.2, a κ-score of 5 and a maximum depth of 100.

Finally, cytoHubba (version 0.1) [30], a Cytoscape plugin, was adopted to explore the network modules and identify hub molecular variables (i.e., DEGs and DELs), defined as those having high correlation in the candidate modules. For each experimental comparison the top 10 variables were identified using the Maximal Clique Centrality method.

In parallel, for each experimental comparison the Regulatory Impact Factor (RIF) analysis was performed within CeTF v.1.16.0 [31]. This metric evaluates transcripts that exhibit significant divergence in their average expression and correlations with highly responsive target genes (RIF1), as well as those showing substantial changes in their patterns of association or functional influence over the expression of target genes (RIF2). The lncRNAs whose RIF1 and RIF2 scores that fell within the top 5% were deemed significant for this analysis and were labelled accordingly. A schematic workflow of the bioinformatic pipeline adopted is reported in Figure S1.

## Results

### Animals’ data

The 31 cows enrolled in this study had an average of 235 (± 78) DIM and an average parity of 2.5 (range 2–5), with most animals being in their second parity. Milk yield averaged 26.97 (± 9.01) kg/d. Milk had 2.19% ± 0.74% of fat, 3.49% ± 0.29% of protein, 2.73% ± 0.27% of casein, and 4.51% ± 0.44% of lactose. Descriptive statistics are reported in Table S1 and more information about milk composition are reported in Bisutti et al. [15].

### Sequencing and alignment

Following quality trimming and adaptor removal, each sample yielded an average of 119 ± 49.8 million reads. The alignment with the bovine genome resulted on an average of 91.92% reads (range: 84.0%–95.2%). Among the mapped reads, 85.69% (range: 77.8%–89.7%) were uniquely mapped, while 6.24% (range: 4.9%–7.6%) exhibited multiple mapping (Table S2).

### LncRNA identification

**Fig. 1:**
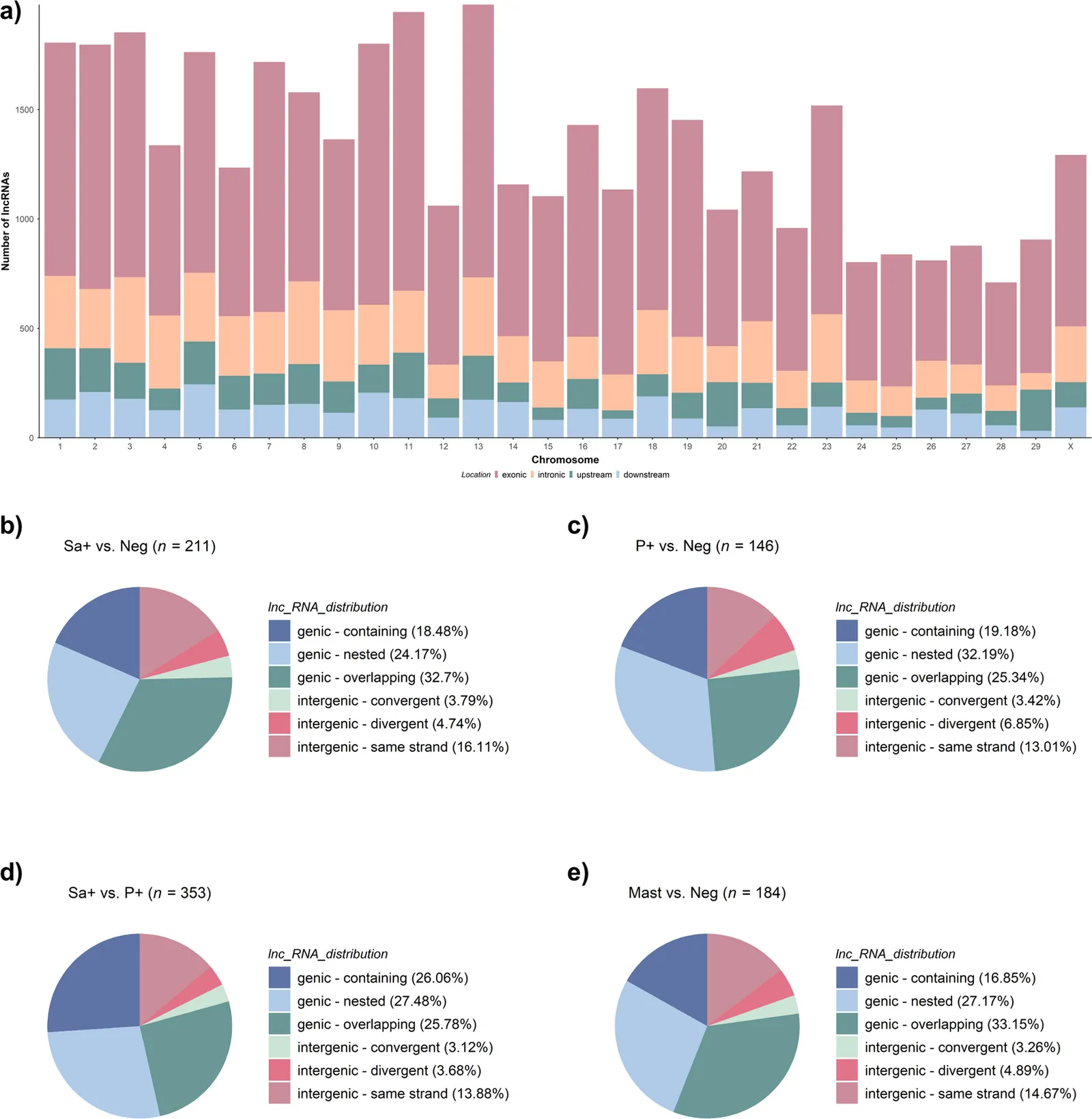
Genomic location and distribution of the identified lncRNAs across the different experimental groups. **a** Chromosomic distribution of the long non-coding RNAs. **b** lncRNAs genomic distribution in Sa+ vs. Neg. **c** lncRNAs genomic distribution in P+ vs. Neg. **d** lncRNAs genomic distribution in Sa+ vs. P+. **e** Mast vs. Neg animals, regardless of the pathogen

First, 2,463,167 transcripts were obtained assembling the 31 samples using Cuffcompare, which included all potential RNA types, such as protein-coding genes, novel genes, lncRNAs and pseudogenes. Of these, 53.5% transcripts have class codes ‘i’, ‘u’, ‘x’, representing non-coding transcripts. This assembled transcript was given to FEELnc tool, which applied a filter pipeline to remove protein-coding, pseudogene, and miRNA, and capture transcripts with a minimal size of 200 bp. A total of 40,586 transcript harboring lncRNAs were identified. Among them, 13,840 (34%) were previously annotated in the bovine reference genome, while the remaining 26,746 (66%) were considered novel. To further characterize the newly identified lncRNAs, we evaluated the structural features of the novel transcripts. These transcripts showed a median length of 2,587 bp (mean = 4,695 bp), a median exon number of 2 (mean = 2.61; range = 2–16 exons), and a median GC content of 46.35% (mean = 47.09%). Overall, these structural characteristics are consistent with those commonly reported for mammalian lncRNAs [4, 5, 8]. Figure 1a depicts distribution and location of the identified lncRNA across the bovine genome. Specifically, 3,885 lncRNAs (9.5%) were located in upstream intergenic regions, 3,887 (9.5%) in downstream intergenic regions, 7,471 (18%) within introns, and 25,343 (62.5%) within exons. Among all chromosomes, BTA3, BTA11, and BTA13 contained the largest numbers of lncRNAs, with 1,980, 1,946, and 1,853 transcripts, respectively. BTA1 and BTA3 contained the highest number of novel lncRNA (*n* = 1,280 and 1,258, respectively).

### Differential expression analysis of lncRNAs

**Fig. 2:**
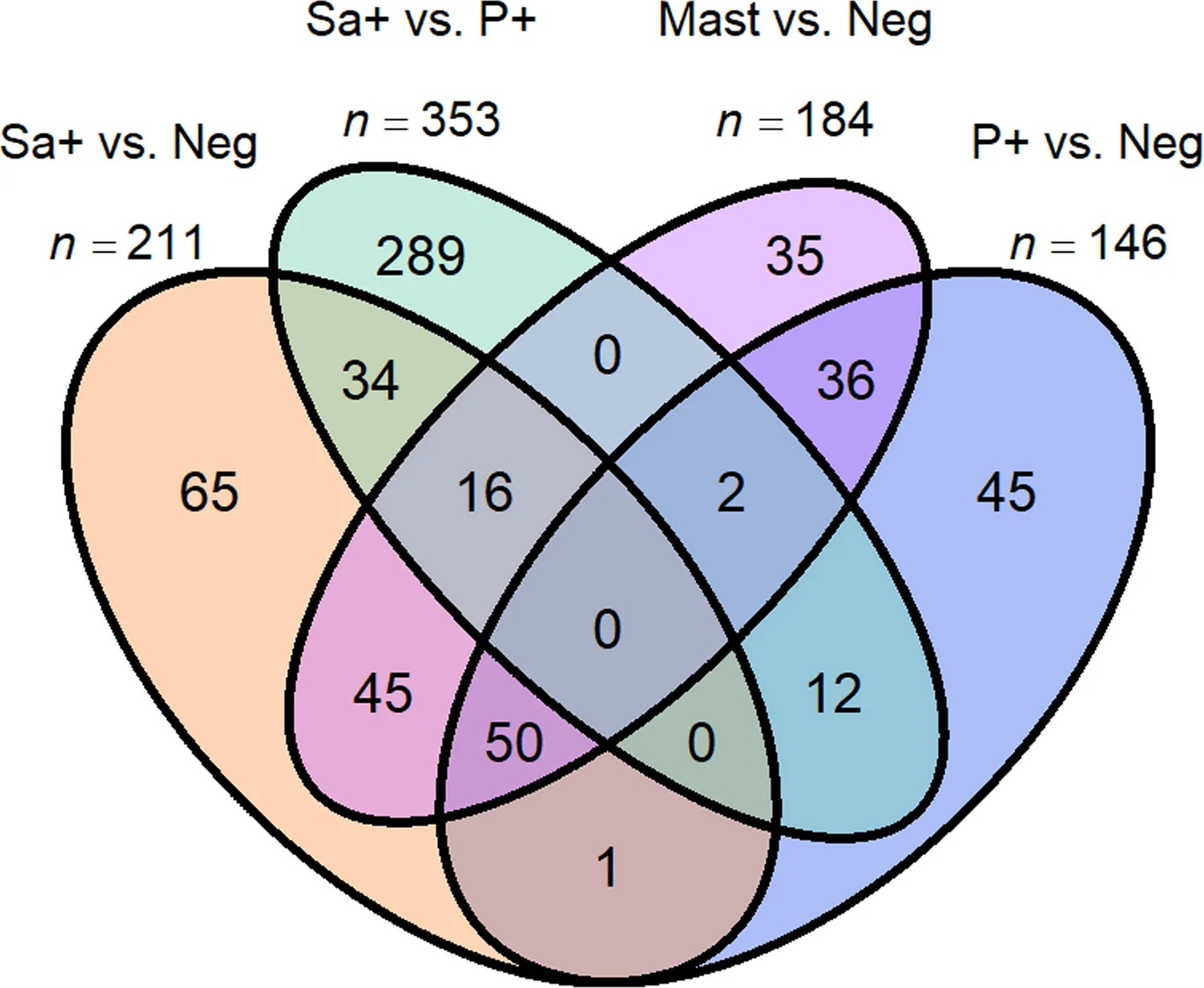
Distribution of DELs across the four experimental comparisons. Sa+: animals naturally infected by *S. agalactiae*. P+: animals naturally infected by *Prototheca* spp. Pos: animals with infection, regardless of the pathogen

The DEGs obtained with the differential analysis for each experimental comparison are reported in Table S3. The DELs were defined by overlapping the DETs identified through transcript-level DESeq2 analysis with the annotated set of putative lncRNAs predicted by FEELnc. A total of 211 DELs were identified for Sa+ vs. Neg, 146 DELs for P+ vs. Neg, 353 DELs for Sa+ vs. P+, and 184 DELs between Mast vs. Neg cows (Table S4). Figure 2 displays the distribution of DELs across the 4 comparisons, showing the extent of overlap among infection-specific, and shared lncRNA responses.

Across all the comparisons, the majority of DELs were localized within genic regions. While minor variations in subtype proportions were observed across conditions, particularly between *Prototheca* spp. and *S. agalactiae* infections, the overall distribution patterns remained largely consistent. Genic-overlapping and genic-nested subtypes consistently represented the largest proportions, ranging from 25.34% to 33.15% and 24.17% to 32.19%, respectively. Genic-containing transcripts were also prevalent, contributing between 16.85% and 26.06% depending on the condition. Intergenic DELs comprised a smaller fraction of each dataset. Among these, the same strand subtype was the most frequent (13.01%–16.11%), while divergent and convergent subtypes were less common, each accounting for less than 7% of transcripts. The classification of the 4 comparisons and the top 10 DELs for each comparison are shown in Fig. 1b–e, and Table 1.

**Table 1:** Top 10 uniquely differentially expressed lncRNAs specific to each pairwise group comparison, with their predicted interaction

lncRNA ID	Position	Comparison	Transcript ID	logFC	FDR	Type	Subtype	Location	Predicted interaction
TCONS_00388494	25:15665481–15666341	Sa+ vs. Neg	-	2.30	0.0043	Genic	Nested	Intronic	*XYLT1*
TCONS_00634106	9:13162781–13174,631	Sa+ vs. Neg	-	−2.18	0.0011	Intergenic	Divergent	Upstream	*SLC17A5*
TCONS_00637681	9:91803635–91821892	Sa+ vs. Neg	-	2.01	0.0070	Genic	Nested	Exonic	*TIAM2*
TCONS_00546151	6:15668131–15672046	Sa+ vs. Neg	-	−1.87	0.0084	Genic	Nested	Intronic	*MCUB*
TCONS_00373638	24:57686800–57701227	Sa+ vs. Neg	-	1.87	0.0025	Genic	Nested	Exonic	*ALPK2*
TCONS_00229865	18:23881889–23885537	Sa+ vs. Neg	-	1.80	0.0037	Intergenic	Same_strand	Upstream	*SLC6A2*
TCONS_00292060	2:69373030–69401852	Sa+ vs. Neg	-	−1.68	0.0029	Intergenic	Same_strand	Ddownstream	*ENSBTAT00000130974*
TCONS_00627470	9:86349660–86381194	Sa+ vs. Neg	ENSBTAT00000115728	−1.61	0.0022	Intergenic	Convergent	Downstream	*UST*
TCONS_00535000	5:70576179–70592820	Sa+ vs. Neg	-	1.54	0.0053	Genic	Overlapping	Exonic	*ABTB3*
TCONS_00130780	13:13067957–13082070	Sa+ vs. Neg	-	−1.42	0.0017	Genic	Nested	Intronic	*CELF2*
TCONS_00638308	9:100887152–100891271	P+ vs. Neg	-	−2.22	0.0040	Genic	Nested	Intronic	*PDE10A*
TCONS_00116144	13:78085191–78095100	P+ vs. Neg	ENSBTAT00000118593	−1.98	0.0008	Genic	Containing	Exonic	*ENSBTAT00000118593*
TCONS_00638309	9:100954923–100960990	P+ vs. Neg	-	−1.89	0.0091	Genic	Nested	Intronic	*PDE10A*
TCONS_00360423	23:48917155–48919187	P+ vs. Neg	-	−1.69	0.0060	Intergenic	Same_strand	Downstream	*F13A1*
TCONS_00106078	12:86870605–86879986	P+ vs. Neg	-	−1.65	0.0027	Intergenic	Convergent	Downstream	*ENSBTAT00000105840*
TCONS_00593149	7:53119188–53126859	P+ vs. Neg	-	−1.57	0.0047	Intergenic	Same_strand	Upstream	*NDFIP1*
TCONS_00210703	17:46270538–46440492	P+ vs. Neg	-	−1.54	0.0032	Genic	Overlapping	Exonic	*RIMBP2*
TCONS_00524315	5:62884670–62888055	P+ vs. Neg	-	−1.53	0.0045	Intergenic	Convergent	Downstream	*ANKS1B*
TCONS_00574147	7:95900746–95910357	P+ vs. Neg	-	−1.44	0.0006	Intergenic	Divergent	Upstream	*CAST*
TCONS_00205206	17:46445471–46453169	P+ vs. Neg	-	−1.39	0.0021	Genic	Nested	Intronic	*RIMBP2*
TCONS_00646689	1:251200–317052	Mast vs. Neg	-	4.29	0.0025	Genic	Overlapping	Intronic	*ENSBTAT00000119326-lncRNA*
TCONS_00646234	1:27537–300889	Mast vs. Neg	-	3.73	0.0053	Genic	Overlapping	Intronic	*ENSBTAT00000069831*
TCONS_00647213	1:646234–677893	Mast vs. Neg	-	2.31	0.0057	Genic	Containing	Exonic	*ENSBTAT00000074414-lncRNA*
TCONS_00671427	X:16296964–16310408	Mast vs. Neg	-	2.04	0.0026	Genic	Nested	Intronic	*MBNL3*
TCONS_00580083	7:69445074–69454129	Mast vs. Neg	-	1.68	0.0055	Intergenic	Divergent	Upstream	*CLINTA1*
TCONS_00168356	15:17394941–17399263	Mast vs. Neg	-	1.52	0.0046	Genic	Nested	Intronic	*ENSBTAT00000068370*
TCONS_00364041	23:25672675–25677891	Mast vs. Neg	-	1.47	0.0063	Genic	Overlapping	Exonic	*ENSBTAT00000064718*
TCONS_00163823	15:74057685–74059892	Mast vs. Neg	-	−1.26	0.0070	Intergenic	Same_strand	Downstream	*ALKBH3*
TCONS_00573847	7:99054775–99056830	Mast vs. Neg	-	−1.24	0.0074	Genic	Nested	Intronic	*ENSBTAT00000102698*
TCONS_00292452	2:122129106–122135524	Mast vs. Neg	-	−1.22	0.0066	Genic	Overlapping	Intronic	*ENSBTAT00000092420-lncRNA*
TCONS_00106358	12:84552011–84560553	Sa+ vs. P+	ENSBTAT00000091705	3.48	0.0007	Intergenic	Divergent	Upstream	*ENSBTAT00000119947-lncRNA*
TCONS_00123043	13:18972551–18,976,436	Sa+ vs. P+	-	2.94	0.0000	Genic	Nested	Intronic	*PARD3*
TCONS_00578761	7: 11116620- 11223288	Sa+ vs. P+	-	2.69	0.0000	Intergenic	Convergent	Downstream	*ADGRE3*
TCONS_00591870	7: 71769123- 71876832	Sa+ vs. P+	-	2.62	0.0006	Genic	Overlapping	Exonic	*CCNJL*
TCONS_00290440	2:118665827–118677557	Sa+ vs. P+	-	2.51	0.0003	Genic	Overlapping	Exonic	*ITM2C*
TCONS_00413097	27:37056099–37059844	Sa+ vs. P+	-	2.51	0.0013	Genic	Nested	Exonic	*PLAT*
TCONS_00090519	11:6457245–6459821	Sa+ vs. P+	ENSBTAT00000107248	2.49	0.0000	Intergenic	Same_strand	Downstream	*ENSBTAT00000106020-lncRNA*
TCONS_00237574	18:11204023–11220388	Sa+ vs. P+	-	2.30	0.0001	Intergenic	Divergent	Upstream	*CIBAR2*
TCONS_00128066	13:39091589–39117201	Sa+ vs. P+	-	2.29	0.0013	Genic	Nested	Intronic	*SLC24A3*
TCONS_00414576	27:37218335–37221815	Sa+ vs. P+	-	2.28	0.0001	Genic	Overlapping	Exonic	*DKK4*

Sa+: Animals naturally infected by *S. agalactiae*; P+: Animals naturally infected by *Prototheca* spp.; Pos: Animals with in regardless of the pathogen

### QTL annotation and enrichment analysis

A total of 1,197, 712, and 2,110 annotated QTLs were identified within a 2 kb window (1 kb upstream and downstream) of the DELs partner genes from the Sa+ vs. Neg, P+ vs. Neg, and Sa+ vs. P+ comparisons, respectively.

Across all the comparisons, most partner genes were co-localized with the *Milk* QTLs class, accounting for 57.64% for Sa+ vs. Neg, 36.24% for P+ vs. Neg, and 71.86% for Sa+ vs. P+.

**Fig. 3:**
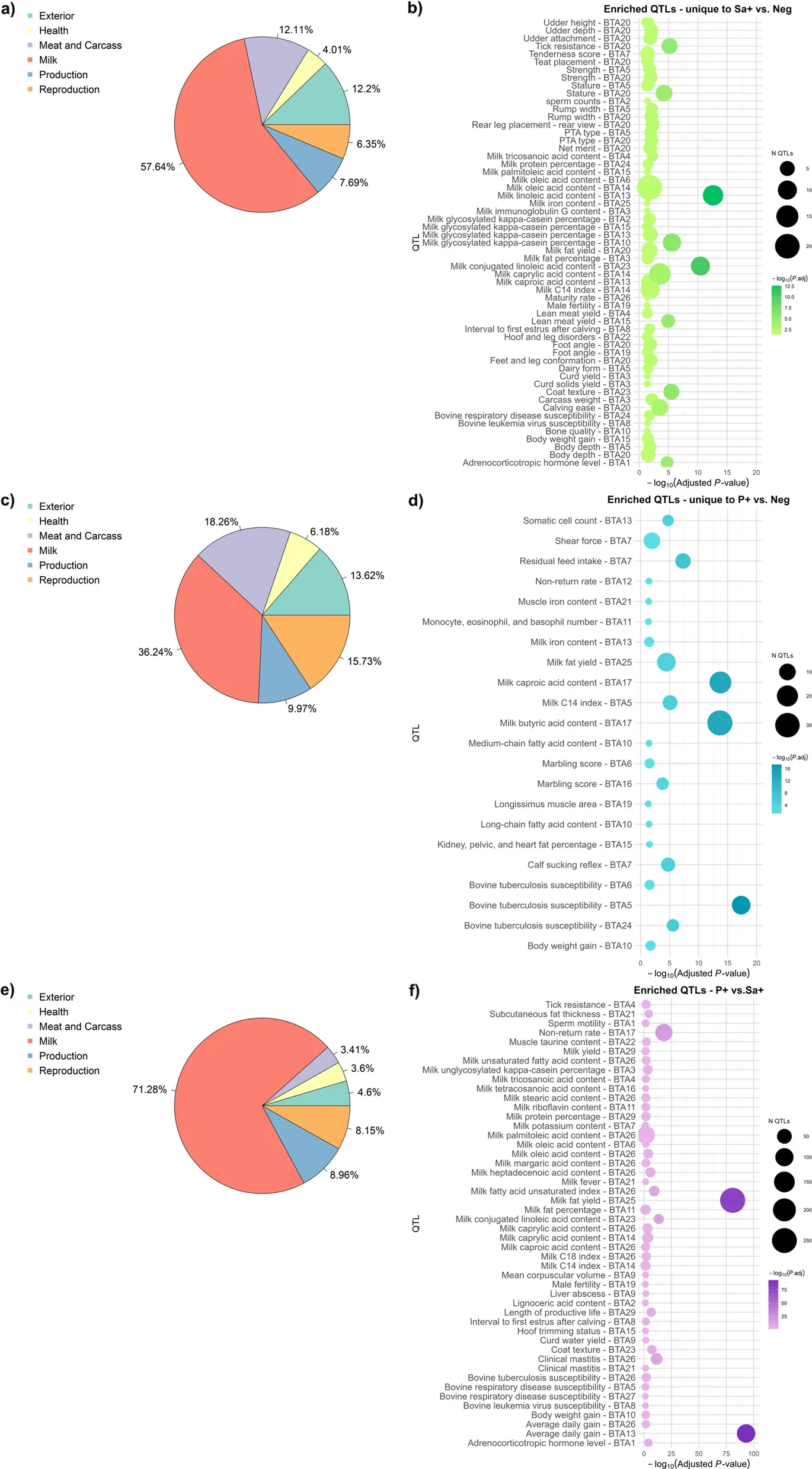
QTLs distribution and enrichment of DELs partner genes. **a** Percentages of each QTL type from the annotation analysis in the Sa+ vs. Neg. comparison. **b** Bubble plot of the QTL enrichment results for the enriched QTLs identified using the partner genes of the 211 DELs. **c** Percentages of each QTL type from the annotation analysis in the P+ vs. Neg. comparison. **d** Bubble plot of the QTL enrichment results for the enriched QTLs identified using the partner genes of the 146 DELs. **e** Percentages of each QTL type from the annotation analysis in the Sa+ vs. P+ comparison. **f** Bubble plot of the QTL enrichment results for the enriched QTLs identified using the partner genes of the 353 DELs. Sa+: animals naturally infected by *S. agalactiae*. P+: animals naturally infected by *Prototheca* spp.

In Sa+ vs. Neg comparison (Fig. 3a), the next most annotated QTLs classes were *Meat and Carcass* (18.26%) and *Reproduction* (15.37%), while for P+ vs. Neg (Fig. 3c), the following classes were Exterior (12.2%) and *Meat and Carcass* (12.11%). When examining partner genes from Sa+ vs. P+ comparison, the second and the third most represented QTLs classes were *Production* and *Reproduction* (8.15%; Fig. 3e). The *Health* QTL class consistently showed the lowest proportions across comparisons, with 4.01% in Sa+ vs. Neg, 6.18% in P+ vs. Neg, and 3.60% in Sa+ vs. P+. The list of QTLs specifically enriched in the Sa+ vs. Neg comparison is shown in Fig. 3b. Notably, a significant enrichment of traits related to the Milk QTL class was observed in this comparison (FDR < 0.05). The enriched QTLs were primarily associated with milk fat and fatty acid composition, as well as traits affecting milk coagulation properties, including milk protein percentage (BTA24), kappa-casein percentage (BTA2, BTA10, BTA13 and BTA15), curd yield, and curd solids (BTA3). In addition, QTLs related to immunoglobulin G content (BTA3) and udder conformation, such as udder height, depth, and attachment (BTA20), were also enriched.

**Fig. 4:**
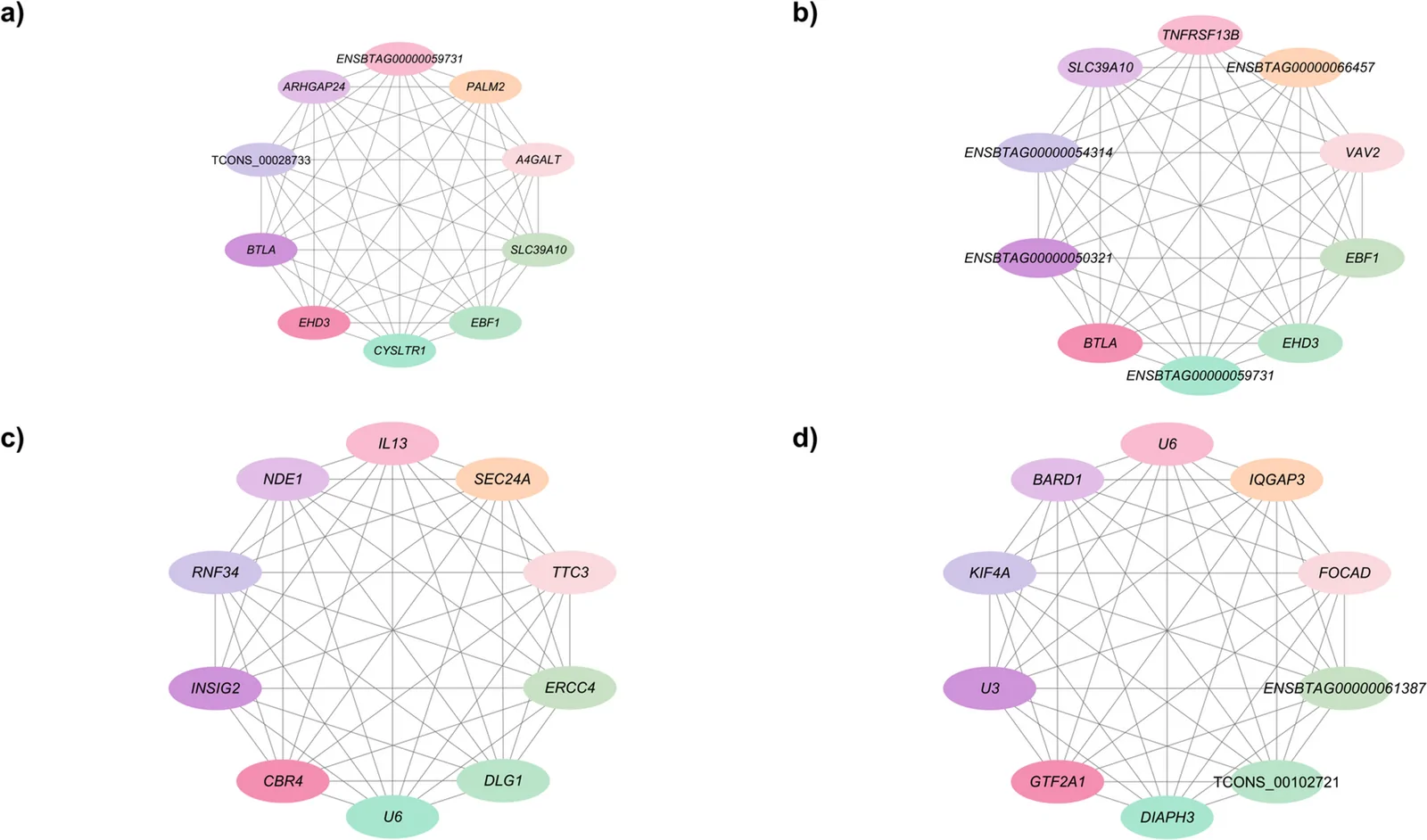
CytoHubba top 10 hub variables according to the Maximal Clique Centrality method for **a** Sa+ groups (Sa+ vs. Neg), **b** P+ group (P+ vs. Neg), **c** Sa+ group (Sa+ vs. P+), and **d** P+ group (Sa+ vs. P+). The highest ranked variable is reported at the bottom of the plot following a clockwise trend

Similarly, in the P+ vs. Neg comparison, enrichment of milk-related QTLs, primarily associated with milk fat and fatty acid composition were observed (Fig. 3d). Interestingly, QTLs for somatic cell count (BTA13), as well as for monocyte, eosinophil, and basophil numbers (BTA11), were also enriched. Additionally, QTLs related to residual feed intake (BTA7) were identified (Fig. 3f). Finally, when comparing Sa+ vs. P+, we found the enrichment of QTL related to clinical mastitis (BTA21 and BTA26; Fig. 4b).

### DELs and DEGs regulatory mechanisms

The PCIT algorithm identified a total of 176,399 and 154,700 molecular traits for the Sa+ vs. Neg samples, respectively, that were reduced to 799 and 933 after filtering for *r* > |0.90|. Concerning P+ vs. Neg samples, we obtained 127,386 and 118,334 traits, respectively (486 and 715 after filtering for *r* > |0.90|). Finally, when comparing the two pathogens, we obtained 441,829 and 320,327 traits for Sa+ and P+ positive samples, respectively (1,220 and 1,099 with *r* > |0.90|). Detailed information of the PCIT outcome for all the experimental comparisons are reported in Table S5 (Sa+ vs. Neg), Table S6 (P+ vs. Neg) and Table S7 (Sa+ vs. P+).

To assess the relationships between the DELs and DEGs we displayed the outcome of the PCIT analysis with Cytoscape. Figure S2 reports the interaction network of the PCIT outcome of the Sa+ infected animals, within the Sa+ vs. Neg comparison. The network consisted of 799 nodes and 4,650 edges. The analysis of the network topological parameters (e.g., closeness centrality and betweenness centrality) highlighted a potential regulatory role of TCONS_00090030 and *LARS1*, while *U6*, *SLC39A10, EBF1* and *CYSLTR1* were the nodes with the highest degree of interactions (145, 115, 110, and 100, respectively). Figure S3 displays the P+ network within the P+ vs. Neg animals’ comparison, consisting of 486 nodes and 3,691 edges. The analysis of the network topological parameters highlighted a potential regulatory role of *ENSBTAG00000069916*, *ENSBTAG00000060444* and *IL21*, while *BANK1, TNFRSF13B* were the genes with the highest degree of interactions (139 and 116, respectively). Moreover, as previously observed for the Sa+ network, *EBF1*, *U6* and *SLC39A10* displayed 142, 133, and 130 interactions, respectively, with other traits in the network, hence confirming their potentially crucial role. Figure S4 shows the Sa+ network, which accounted for 1,220 nodes and 32,550 edges. According to the closeness centrality and betweenness centrality parameters, the *U6* and *ENSBTAG00000061387* resulted to be the ones having a potentially insightful regulatory role. Moreover, *U6*, *CBR4*, *CACUL1* and *IL13* genes resulted to be ones having the highest number of significant interactions (642, 488, 404 and 348 edges, respectively), while TCONS_00326333, TCONS_00085341 and TCONS_00325659 were the DELs having the highest number of connections (207, 180 and 169, respectively). Finally, Figure S5 displays the P+ network generated by the PCIT outcome within the Sa+ vs. P+ comparison, which consists of 1,099 nodes and 10,447 edges. The analysis of the network topological parameters (e.g., closeness centrality and betweenness centrality), highlighted that the novel lncRNA TCONS_00343408, the *SMARCA2* and *PLEKHA8,* involved in the chromatin-remodelling processes and ceramide/glycosphingolipid transport, respectively, might have a significant influence on immune pathways within the regulatory network. When ranking the nodes according to their degree (number of significant interactions), we found *DIAPH3*, *KIF4A* and *BDH2* at the top of the list with 18, 16 and 16 edges, respectively. Table 2 displays the key pairs of DEGs and DELs, selected by prioritizing the network nodes with the highest number of interactions and, subsequently, the DEG–DEL pairs showing the strongest correlations.

**Table 2:** Top five DEG–DEL co-expression pairs identified by PCIT analysis

DEG	DEL	Correlation (*r*)	Comparison	Network
*U6*	TCONS_00585426	0.953	Sa+ vs. Neg	Sa+
*SLC39A10*	TCONS_00028733	0.981	Sa+ vs. Neg	Sa+
*EBF1*	TCONS_00579549	0.978	Sa+ vs. Neg	Sa+
*CYSLTR1*	TCONS_00028733	0.986	Sa+ vs. Neg	Sa+
*BLK*	TCONS_00640342	0.962	Sa+ vs. Neg	Sa+
*EBF1*	TCONS_00579549	0.991	P+ vs. Neg	P+
*BANK1*	TCONS_00558074	0.997	P+ vs. Neg	P+
*ENSBTAG00000059731*	TCONS_00579549	0.987	P+ vs. Neg	P+
*U6*	TCONS_00166316	0.960	P+ vs. Neg	P+
*SLC39A10*	TCONS_00579549	0.989	P+ vs. Neg	P+
*U6*	TCONS_00329753	0.973	Sa+ vs. P+	Sa+
*CBR4*	TCONS_00085341	0.986	Sa+ vs. P+	Sa+
*ENSBTAG00000061387*	TCONS_00587286	0.973	Sa+ vs. P+	Sa+
*CACUL1*	TCONS_00520317	0.976	Sa+ vs. P+	Sa+
*FOXK2*	TCONS_00326333	0.990	Sa+ vs. P+	Sa+
*U6*	TCONS_00534964	0.960	Sa+ vs. P+	P+
*ENSBTAG00000061387*	TCONS_00102721	0.986	Sa+ vs. P+	P+
*DIAPH3*	TCONS_00102721	0.987	Sa+ vs. P+	P+
*SCN8A*	TCONS_00102721	0.980	Sa+ vs. P+	P+
*ENSBTAG00000057979*	TCONS_00448157	0.969	Sa+ vs. P+	P+

Sa+: Animals naturally infected by *S. agalactiae*; P+: Animals naturally infected by *Prototheca* spp. Pairs were selected from each PCIT network by considering hub variables with the highest degree of connectivity and the strongest DEG–DEL correlations

To further investigate the organization of the co-expression networks, MCODE analysis was performed to identify densely connected subnetworks within each co-expression network. However, no significant functional enrichment was detected for the identified modules. For completeness, the module composition and network visualizations are reported in Fig. S1–S4 and Tables S5–S7.

The first 10 hub variables selected from cytoHubba, for each experimental comparison are reported in Fig. 4. For Sa+ vs. Neg, the algorithm identified as potentially most informative variables for *S. agalactiae* infection 9 genes (*ARHGAP24*, *BTLA*, *EHD3*, *CYSLTR1*, *EBF1*, *SLC39A10, A4GALT, PALM2,* and *ENSBTAG00000059731)* and the novel lncRNA TCONS_00028733, while for P+ vs. Neg comparison, no lncRNAs were selected among the most informative variables capable of discriminating the infection status, but only genes (i.e., *TNFRSF13B*, *SLC39A10*, *BTLA, EHD3* and *EBF1*, and *VAV2*). Whitin the Sa+ vs. P+ comparison, cytoHubba identified at top 10 variables for Sa+ infection *U6*, *DLG1*, *ERCC4*, *TTC3*, *SEC24A*, *IL13*, *NDE1*, *RNF34*, *INSIG2* and *CBR4*, while for P+ infection, one lncRNA was identified as one of the top regulators for the infection (i.e., TCONS_00102721) together with 9 genes (i.e., *DIAPH3, FOCAD*, *IQGAP3*, *U6*, *BARD1*, *KIF4A*, *U3* and *GTF2A1*).

The RIF analysis was conducted separately for each experimental group. For Sa+ vs. Neg animals, 22 lncRNAs ranked within the top 5% according to RIF1 and RIF2 scores (RIF1 > 2.29 and RIF2 > 1.77, respectively). Of these, 11 were also identified in the Sa+ PCIT network. Concerning P+ vs. Neg animals, 13 of the 123 evaluated lncRNAs ranked within the top 5% according to RIF1 and RIF2 scores, using thresholds of RIF1 > 2.21 and RIF2 > 1.40. Of these 13 lncRNAs, 12 were also identified in the corresponding P+ PCIT network. Finally, for the Sa+ vs. P+ comparison, 34 lncRNAs were ranked within the top 5% according to both RIF1 and RIF2 scores (RIF1 > 2.04 and RIF2 > |1.99|). Because PCIT generated separate co-expression networks for the Sa+ and P+ groups, 26 lncRNAs were also identified in the Sa+-specific PCIT network and 13 in the P+-specific PCIT network.

## Discussion

To the best of our knowledge, this is the first study that evaluated, through an integrated mRNA and lncRNAs approach, the molecular variation in response to the infection of two phylogenetically distant pathogens: *S. agalactiae*, a Gram-positive bacterium, and *Prototheca* spp., a microscopic alga. *Prototheca* spp., has been considered until recently only an emerging mastitis agent, but is now starting to be recognized as a non-negligible problem due to its increasing prevalence worldwide, such as USA, Canada, Belgium, Brazil, Italy and other countries [32] despite its mechanism of action still being poorly understood. The estimated prevalence on the Italian territory has been reported as 11.2% [33], while outbreaks in Brazil have reached peaks of 36.49% of animals positive to this alga, highlighting the potential impact of this pathogen [32].

The DE analysis of the transcripts, combined with the FEELnc tool, allowed the identification of DELs across all the experimental comparisons. In accordance with the work conducted by Asselstine et al. [12], approximately 78% of the total of DELs identified across all the experimental comparisons were classified as novel, meaning that they were not previously annotated for their gene name or length in the bovine reference genome. In our study, most of the identified DE lncRNAs were classified as genic (76%, 77%, 80% and 58% for Sa+ vs. Neg, P+ vs. Neg, Sa+ vs. P+, and Mast vs. Neg, respectively), while the remaining proportion was found to be intergenic. These results differ from those reported by Asselstine et al. [12], who observed that lncRNAs identified in milk somatic cells from mastitic animals were located mostly within intergenic regions. This differing proportion of genic vs. intergenic lncRNAs could be influenced by sequencing depth and annotation quality [34] but we could also hypothesize that the type of infection (clinical vs. subclinical) and/or the invading pathogen may contribute to this pattern.

### *S. agalactiae* and immune-metabolic pathways regulation

Within the Sa+ vs. Neg comparison the most over-expressed was the novel lncRNA TCONS_00388494 (logFC = 2.30, FDR = 0.0043). This genic lncRNA was predicted to interact with the *XYLT1* gene as it overlaps its intronic region. The *XYLT1* gene encodes for the Xylosyltranferase 1, a key enzyme that catalyzes the initial step in proteoglycan biosynthesis and has been shown to be upregulated in human fibroblasts under fibrotic conditions [35]. Fibroblasts do not only play a role in the synthesis of the extracellular matrix, but also contribute to the activation and regulation of immune response by acting as immune sentinel cells upon the detection of pathological stimuli [36]. Mastitis is characterized by an inflammatory process driven by tissue injury. Although direct evidence in cattle is currently lacking, findings from other biological systems may point to a potential role of this gene in the regulation of inflammatory responses.

Another DEL upregulated in Sa+ infection was the intergenic TCONS_00578761 which, based on its downstream position, was predicted to interact with the *ADGRE3* (Adhesion G Protein-Coupled Receptor E3) gene. This gene is highly expressed in granulocytes, neutrophils and eosinophils [37] and might play a role in myeloid-myeloid interactions during immune and inflammatory responses. Besides its immune function, *ADGRE3* participates in acetate utilization to produce acetyl-CoA for lipid synthesis or energy metabolism [38]. Interestingly, when considering the QTLs uniquely enriched in the Sa+ vs. Neg comparison, we observed that almost half of the lncRNA partner genes clustered within milk-related QTL regions, especially those associated with fat and fatty acid composition. While QTL co-localization alone does not necessarily imply a functional relationship, this pattern is in line with observations from our previous gene expression study, in which *S. agalactiae* infection appeared to trigger the activation of metabolic processes, such as those linked to fatty acid metabolism [15, 39]. In addition, among the key DEG–DEL pairs identified (Table 2), we observed strong positive correlations between TCONS_00326333 and *FOXK2* (Forkhead box K2) and between TCONS_00085341 and *CBR4* (Carbonyl reductase 4). Notably, *FOXK2* has been reported to participate in the regulation of fatty acid metabolism [40] and has been identified among key genes associated with bovine clinical ketosis [41]. Similarly, *CBR4* encodes a mitochondrial protein belonging to a family involved in fatty acid biosynthetic processes [39].

Out of the 11 DELs identified by both PCIT and RIF analysis of the Sa+ network (Sa+ vs. Neg comparison), 3 of them (TCONS_00298220, TCONS_00149802, TCONS_00149801) displayed a high number of interactions with the other variables in the co-expression network. Specifically, TCONS_00149801 and TCONS_00149802 were found to interact not only with each other, but also with *LYST* and *PTKB2,* which are involved in inflammatory and immune processes. In the study conducted by Riquier et al. [42] on the role of lncRNAs in the immunity of wild bats, *LYST* (lysosomal trafficking regulator) was identified as a gene associated with numerous lncRNAs. While these findings derive from a different species, they highlight LYST as a potentially relevant candidate for future investigations of lncRNA-gene interactions in cattle.

When computing the top 10 hub variables for the discrimination of *S. agalactiae* infection, *CYSLTR1* (cysteinyl leukotriene receptor 1) resulted to be the highest ranked gene. This gene encodes a G protein-coupled receptor for cysteinyl leukotrienes, which are highly potent lipid inflammatory mediators [43]. While initially recognized as mediators in acute respiratory disease such as asthma, they have recently been to play a driving role in the progression of chronic inflammatory diseases in humans [44]. Although this evidence originates from human inflammatory diseases and not from bovine mastitis, it provides a rationale to further investigate the potential role of *CYSLTR1* in thein inflammatory response associated with mastitis.

### *Prototheca* spp. and immune response regulation

Among the top 10 uniquely expressed lncRNAs within the P+ vs. Neg comparison, TCONS_00638308 and TCONS_00638309 (logFC = −2.22 and −1.89, respectively) were both predicted to interact with *PDE10A* gene*.* This gene encodes the phosphodiesterase 10 A (PDE10A), an enzyme that hydrolyzes cAMP and/or cGMP and is therefore involved in cyclic nucleotide homeostasis. *PDE10A* was implicated in adaptive immune response, particularly related to T lymphocyte activation and modulation, in a gene set enrichment analysis of SNP data from cattle affected by bovine respiratory disease [45]. Moreover, Feng et al. [46] reported this gene enriched in pathways associated to environmental response and immunity in an endangered Chinese cattle breed. While these findings derive from different biological contexts, they could pave the way for supporting potential involvement of *PDE10A* and its associated lncRNAs in the regulation of bovine mastitis. the potential involvement of *PDE10A* in regulating the host immune response during bovine mastitis.

Enrichment in milk QTL regions was also observed in the P+ vs. Neg comparison, but to a lesser extent. These results are consistent with previous studies reporting that enriched QTLs associated with DEL target genes are mainly linked with milk production traits [12, 47, 48]. However, in the uniquely enriched QTLs within the P+ vs. Neg comparison, we observed associations mostly with immune response traits, such as somatic cell count and monocyte, eosinophil and basophil number, rather than traits linked to milk production, composition and technological properties, as observed in *S. agalactiae* infection. This slight divergence suggests that the host may activate distinct pathways depending on the infecting pathogen, reflecting differences in host–pathogen interaction mechanisms and in the resulting physiological impact on the mammary gland. Overall, these findings should be interpreted as exploratory and further validation is required to clarify potential regulatory links.

In the P+ co-expression network built within the P+ vs. Neg comparison, of the 12 DELs commonly shared by the PCIT and RIF analysis, TCONS_00028733, TCONS_00579552 and TCONS_00588162 showed a high number of interactions not only with each other, but also with several genes linked to B cells signaling (e.g., *CD79B*, *FCRL3, BANK1, MS4A1, TNFRSF13B*) and innate immune response (e.g., *TLR10*, *CYSLTR1*, *BTLA*, *CD274*, *ITGA1*), suggesting their potential association with such biological processes. To the best of our knowledge, no studies up to date have identified lncRNAs targeting any of these genes in cattle or other ruminants. However, Zhang et al. [49] in a study conducted on mouse models, discovered that the lncRNA *Linc00662* regulates the expression of *ITGA1*, a gene involved in focal adhesion pathways, by activating its transcription. Huang et al. [50] investigated the role of the lncRNA small nucleolar RNA host gene 12 (*SNHG12*) in the regulation of cell growth and drug resistance in non-small cell lung cancer. In that context, *SNHG12* facilitated the escape of cancer cells by increasing the expression of *CD274*, which encodes programmed death-ligand 1 (PD-L1), a key immune checkpoint molecule known to block the activation of immune cells and the generation of antibodies and cytokines that dampen the immune response [51]. Although these findings derive from a cancer model and should therefore be interpreted with caution, they point out the broader capacity of lncRNAs to modulate immune-related pathways, including those involved in host–pathogen interactions. *Prototheca* spp. is a pathogenic alga whose mechanism of action is still currently unknown but it is believed to be able to survive or evade host immune response as a mechanism to exert pathology [33]. Within this framework, lncRNAs may potentially contribute to this effect and in the regulation of host immune response with possible implications for the development of targeted interventions.

When considering the most informative cytoHubba variables identified for *Prototheca* infection, *TNFRSF13B* was one of the most informative. The *TNFRSF13B* is a member of TNF receptors and specifically encodes for the transmembrane activator and calcium modulator and cyclophilin ligand interactor which regulates immune cell homeostasis, differentiation and function, it is embedded in a complex network of cytokines [52] and it can serve as a receptor for the B cell activating factor (BAFF). Interestingly, this gene was found up-regulated in meat ewes naturally affected by clinical mastitis [53], suggesting its potential role in bovine models as well. Moreover, previous research conducted by [54] evidenced that *TNFRSF1A* may act as a hub gene in bovine mastitis. While *TNFRSF13B* belongs to the same receptor superfamily, additional evidence is required before drawing conclusions regarding its role in bovine mastitis. Nevertheless, its identification among the most informative variables in the present study supports its consideration as a candidate gene for future investigation.

### Commonly shared regulatory mechanisms between the two types of infection

In previous work in a similar cohort of animals [14] we highlighted the involvement of different leukocyte populations between the two microorganisms, with responses mainly dominated by innate immunity in *S. agalactiae* infections and by adaptive immunity in *Prototheca* spp. infections. In this work, we did not observe marked differences either in the DELs targeted genes, or in the co-expression regulatory mechanisms associated with the two pathogens. This outcome is consistent with findings from previous transcriptomic [15] and DNA methylation [55] studies conducted in the same cohort of animals and may reflect the presence of a core host regulatory response shared between the two pathogens under the specific conditions of this population.

Out of the 34 DELs identified by the PCIT and RIF analyses within the Sa+ vs. P+ comparison, 26 were also present in the Sa+ co-expression network and 13 in the P+ network. Overall, 9 were shared between both networks, suggesting a partial overlapping immune regulatory response, regardless of the pathogen, and highlighting a core set of genes that may contribute to a general host defense mechanism in subclinical mastitis. Interestingly, all these DELs interacted with several genes linked to immunity and inflammatory responses, as well as with spliceosomal *U2*, *U4*, *U5* and *U6* genes. While no direct links with bovine mastitis have been identified for *U4* and *U5*, Lai et al. [56] reported an overexpression of *U2* (i.e., *RNU2*) in milk from cows with clinical mastitis. However, to date, no lncRNAs have been identified targeting this gene. In addition, Asselstine et al. [12] identified in their research on milk somatic cells a novel lncRNA (i.e., *lncRNA_1505.1*) that was predicted to interact with *U6* during mastitis-associated inflammation.

Notably, among the top 10 hub variables, we identified several genes that were commonly shared in both types of infection (i.e., *EBF1*, *BTLA*, *SLC39A10*, *EHD3*), which might indicate that, despite the phylogenetic distance existing between *S. agalactiae* and *Prototheca* spp., core immune response mechanisms may be conserved and activated in response to both pathogens. Indeed, *EBF1*, which encodes for early B cell factor-1 plays a critical role in specifying the B-cell differentiation [57, 58]. In the study conducted by Griffin et al. [59] in human adipocytes the authors demonstrated that *EBF1* was a key component in the positive regulation of metabolic and inflammatory pathways, such as PI3K-AKT, MAPK, and STAT1, suggesting also a potential association with the regulation of NF-κB signaling pathways. Although these findings derive from a different biological context, they suggest that this gene may represent a promising candidate for further investigation in the context of mastitis. B and T lymphocyte attenuator (*BTLA*) encodes for a member of the CD28 immunoglobulin superfamily and is expressed on most lymphocytes and acts as an inhibitor of B and T cells activation and proliferation, functioning as a brake on immune responses [60]. It might therefore be hypothesized that *EBF1* and *BTLA* may contribute to the modulation of inflammatory and adaptive immune responses during mastitis, although their specific roles require further investigation.

Co-expression networks were constructed not only for infected samples but also for negative controls to define the baseline transcriptional organization of the mammary gland in the absence of infection. Interestingly, the networks constructed from negative samples exhibited differences in topology, with limited overlap between them (Table S5 and S6). This highlights the presence of natural variation in baseline co-expression patterns which could be influenced by other factors such as genetics or environmental conditions. Understanding this baseline variability is critical for interpreting the regulatory impact of DELs in infected animals, since the effects of lncRNAs on genes may be modulated by pre-existing differences in the co-expression structure, making regulatory roles context dependent. Future studies should further explore how baseline network connection may influence infection-dependent regulatory mechanisms.

### Limitations

This study presents some limitations that should be considered when interpreting the results. Firstly, working with naturally occurring infections increases the biological relevance of the findings but introduces also biological variability that cannot be fully controlled as the precise stage and duration of infection, are unknown. In addition, because animals were classified based on quarter-level infection status while transcriptomic analyses were performed on milk somatic cells of composite milk, potential dilution effects may have influenced the observed expression patterns. Furthermore, as RNA was extracted from milk somatic cells, whose cellular composition may vary during different stages of infection and/or inflammation, this might have affected the lncRNAs detection and expression profiles.

It should also be noted that lncRNA identification and functional annotation rely largely on computational prediction and co-expression-based inference. In this context, the absence of experimental validation, such as RT-qPCR or functional assays, should be acknowledged. However, the study was designed as a discovery-oriented analysis, as commonly applied in transcriptomic investigations, and validation was beyond its primary scope. The relatively small sample size may also limit the generalizability of the findings, that should therefore be interpreted within an exploratory framework. Further validation, ideally in an independent population, will be valuable to confirm the biological relevance of these findings.

## Conclusions

This study expands current knowledge on the transcriptomic landscape of bovine milk somatic cells during subclinical mastitis by identifying a wide array of lncRNAs, the majority of which represent novel transcripts that may contribute to the host response to intramammary infection. In *S. agalactiae*-infected cows, candidate lncRNAs were mainly associated with genes and QTLs involved in lipid metabolism, milk fat composition, extracellular matrix remodeling, and inflammatory processes (e.g., TCONS_00326333 and TCONS_00085341). In contrast, candidate lncRNA in *Prototheca* spp.-infected cows showed associations linked to immune cell activation, B-cell-related genes, innate immune signaling, and somatic cell count-associated QTLs (e.g., TCONS_00102721). While the lncRNA expression signatures differed between pathogens, network analyses revealed a partial overlap of candidate hub variables (e.g., *EBF1* and TCONS_00579549*)* suggesting the existence of conserved components of the mammary gland response to subclinical intramammary infection. These shared molecular candidates represent promising targets for future molecular validation and functional characterization. Overall, the results suggest that pathogen-specific lncRNA signatures may contribute to distinct molecular pathways underlying the host response to different intramammary pathogens, whereas shared immune-related regulatory hubs may reflect conserved mechanisms of host defense. Together, these findings underscore the complexity of lncRNA-mediated regulation and highlight the need for functional studies to clarify the biological roles of these newly identified lncRNA in the response to bovine intramammary infection.

## Supplementary Information

Additional file 1: Fig. S1. Schematic flow chart of the bioinformatic pipeline.

Additional file 2: Table S1. Descriptive statistics of milk yield, milk composition and udder health traits.

Additional file 3: Table S2. Statistics of the mapped reads.

Additional file 4: Table S3. List of the differentially expressed genes for each experimental comparison.

Additional file 5: Table S4. List of the differentially expressed long non-coding RNA for each experimental comparison.

Additional file 6: Table S5. Results of the PCIT analysis, RIF analysis, and Cytoscape network statistics for the Sa+ vs. Neg experimental group.

Additional file 7: Table S6. Results of the PCIT analysis, RIF analysis, and Cytoscape network statistics for the P+ vs. Neg experimental group.

Additional file 8: Table S7. Results of the PCIT analysis, RIF analysis, and Cytoscape network statistics for the Sa+ vs. P+ experimental group.

Additional file 9: Fig. S2. Sa+ network and module analysis carried out with Cytoscape plugin MCODE. Each node corresponds to a different gene/lncRNA. Nodes belonging to different modules are differently colored. Light grey nodes correspond to the variables that were not assigned to any modules.

Additional file 10: Fig. S3. P+ network and module analysis carried out with Cytoscape plugin MCODE. Each node corresponds to a different gene/lncRNA. Nodes belonging to different modules are differently colored. Light grey nodes correspond to the variables that were not assigned to any modules.

Additional file 11: Fig. S4. Sa+ network and module analysis carried out with Cytoscape plugin MCODE. Each node corresponds to a different gene/lncRNA. Nodes belonging to different modules are differently colored. Light grey nodes correspond to the variables that were not assigned to any modules.

Additional file 12: Fig. S5. P+ network and module analysis carried out with Cytoscape plugin MCODE. Each node corresponds to a different gene/lncRNA. Nodes belonging to different modules are differently colored. Light grey nodes correspond to the variables that were not assigned to any modules.

## Data Availability

The sequencing data of this study were deposited in the National Center for Biotechnology Information’s (NCBI’s) Sequence Read Archive (SRA) under the PRJNA911953 accession number.

## Abbreviations

A4GALT: Alpha 1,4-galactosyltransferase
ADGRE3: Adhesion G protein-coupled receptor E3
ARHGAP24: Rho GTPase activating protein 24
BANK1: B cell scaffold protein with ankyrin repeats 1
BARD1: BRCA1 associated RING domain
BDH2: 3-Hydroxybutyrate dehydrogenase 2
BTLA: B and T lymphocyte associated
CACUL1: CDK2 associated cullin domain
CBR4: Carbonyl reductase 4
CD274: CD274 molecule
CD79B: CD79b molecule
CPS: Coding potential score
CYSLTR1: Cysteinyl leukotriene receptor 1
DEG: Differentially expressed gene
DEL: Differentially expressed long non-coding RNA
DET: Differentially expressed transcripts
DIAPH3: Diaphanous related formin 3
DIM: Days in milk
DLG1: Discs large MAGUK scaffold protein 1
EBF1: EBF transcription factor 1
EHD3: EH domain containing 3
ERCC4: Excision repair 4, endonuclease catalytic subunit
FCRL3: Fc receptor like 3
FDR: False discovery rate
FOCAD: Focadhesin
GTF2A1: General transcription factor IIA subunit 1
IL13: Interleukin 13
IL21: Interleukin 21
INSIG2: Insulin induced gene 2
IQGAP3: IQ motif containing GTPase activating protein 3
ITGA1: Integrin subunit alpha 1
ITM2C: Integral membrane protein 2C
IZSVe: Istituto zooprofilattico sperimentale delle venezie
JAK-STAT: Janus kinase/signal transducers and activators of transcription
KIF4A: Kinesin family member 4A
LARS1: Leucyl-TRNA synthetase 1
lncRNA: Long non-coding RNA
LYST: Lysosomal trafficking regulator
MAPK: Mitogen-activated protein kinases
MCC: Maximal clique centrality
MS4A1: Membrane spanning 4-domains A1
ncRNA: Non-coding RNA
NDE1: NudE neurodevelopment protein 1
NGI: Nuova genetica italiana
NK-кB: Nuclear factor-κB
ORF: Open reading frame
P+: *Prototheca* spp. positive group
PALM2: Paralemmin 2
PCIT: Partial correlation and information theory
PDE10A: Phosphodiesterase 10A
PIM: *Prototheca* spp. isolation medium
PLEKHA8: Pleckstrin homology domain containing A8
PTK2B: Protein tyrosine kinase 2 beta
QTL: Quantitative trait loci
RIF: Regulatory impact factor
RNF34: Ring finger protein 34
rRNA: Ribosomal RNA
Sa+: *Streptococcus* *agalactiae* positive group
SEC24A: SEC24 homolog A, COPII component
sIMI: Subclinical intramammary infection
SLC39A10: Solute carrier family 39 member 10
SMARCA2: SWI/SNF related BAF chromatin remodeling complex subunit ATPase 2
SNHG12: Small nucleolar RNA host gene 12
TLR10: Toll like receptor 10
TNFRSF13B: TNF receptor superfamily member 13B
TNFRSF1A: TNF receptor superfamily member 1A
tRNA: Transfer RNA
TTC3: Tetratricopeptide repeat domain 3
U2: RNA, U2 small nuclear 2
U3: RNA, U4 small nuclear 3
U4: RNA, U4 small nuclear 8
U5: RNA, U4 small nuclear 5
U6: RNA U6, small nuclear 6
VAV2: Vav guanine nucleotide exchange factor 2
XYLT1: Xylosyltransferase 1
